# Total hip arthroplasty with porous tantalum trabecular metal pads in patients with Crowe IV developmental dysplasia of the hip: a midterm followup study

**DOI:** 10.1186/s12891-024-07598-5

**Published:** 2024-07-03

**Authors:** Cheng Yang, Donghai Li, Shuo Sun, Zhouyuan Yang, Pengde Kang

**Affiliations:** Department of Orthopaedics Surgery, West China Hospital, Sichuan University, 37# Wainan Guoxue Road, Chengdu, Sichuan 610041 People’s Republic of China

**Keywords:** Acetabular reconstruction, Porous tantalum pads, Crowe IV DDH, Total hip arthroplasty

## Abstract

**Purpose:**

Crowe IV developmental dysplasia of the hip (DDH) is a catastrophic hip disease. Moreover, obtaining ideal clinical efficacy in conventional total hip arthroplasty (THA) is often difficult. In this study, we aimed to assess the mid-term clinical results of THA with porous tantalum trabecular metal (TM) pads for acetabular reconstruction in the treatment of Crowe IV DDH.

**Methods:**

A cohort of 28 patients (32 hips) diagnosed with Crowe type IV DDH who underwent acetabular reconstruction during THA using TM pads with scheduled follow-up between 2011 and 2018, were included in this study. Eight cases were men and 24 were women, with a mean age of 48.4 years (range, 36–72 years) and a mean follow-up was 74.3 months (range, 42–132 months). All patients underwent acetabular reconstruction using TM pads and total hip replacement with subtrochanteric osteotomy.

**Results:**

At the final follow-up, 28 hips (87.5%) demonstrated mild or no postoperative limping. The Harris Hip Score improved from 58.4 ± 10.6 preoperatively to 85.6 ± 8.9. The mean pain, stiffness, and function scores on the Western Ontario and McMaster University Osteoarthritis index were 86.5 ± 10.2, 87.3 ± 12.4 and 85.4 ± 11.6 respectively. The mean score of patient satisfaction was 90.4 ± 7.6. Additionally, the SF-12 physical summary score was 41.8 ± 5.6 and the SF-12 mental summary score was 51.6 ± 5.4. TM construct survivorship due to all-cause failure was 90.6% at 5 years with 3 hips at risk, 87.5% at 10 years with 4 hips at risk. The survivorship due to failure from aseptic loosening was 96.9% at 5 years with 1hips at risk and 93.75% at 10 years with 2 hips at risk.

**Conclusion:**

This study demonstrated satisfactory mid-term clinical and radiological results with the application of TM pads for acetabular reconstruction combined with THA in patients with Crowe IV DDH.

**Trial registration number:**

ChiCTR1800014526, Date: 18/01/2018.

## Introduction

Type IV developmental dysplasia of the hip (DDH) is one of the most complex hip deformities that requires reconstruction [1]. DDH is the main cause of degenerative arthritis of the hip and valgus deformity of the knee and eventually necessitates total hip arthroplasty (THA) [2–4]. Moreover, THA is regarded as the optimal treatment choice for DDH and is associated with a high rate of functional improvement and pain relief [5–9]. THA has proven to be successful in the reconstruction of advanced DDH with functional impairments [10–12]. However, THA for Crowe IV DDH is technically challenging due to extensive distortions of the native anatomy. Patients with DDH may have a shallow acetabulum, a straight narrow femoral canal, and associated circumferential soft tissue deformities [13].

The distorted anatomy of the acetabulum and proximal femur poses a major challenge during THA [14–16]. Various methods and techniques have been proposed to restore the normal anatomical relations of the distorted hip joint in Crowe IV DDH. Additionally, previous studies have described several strategies to reconstruct the abnormal acetabulum using autogenous femoral head grafts, embedded bone grafts, and porous tantalum cups plus granular bone grafts during THA [16, 17]. However, the optimal treatment remains unclear. For more than a decade, we have used trabecular metal (TM) pads and augmentation techniques for acetabular reconstruction in hip revision or complex THA [18].

However, the application of TM pads in the treatment of Crowe type IV DDH has not been well reported. This study aimed to evaluate the clinical efficacy of mid-term THA combined with porous TM pads for acetabular reconstruction in patients with Crowe type IV DDH.

## Materials and methods

After obtaining approval from the Institutional Review Board of West China Hpsital, we conducted a retrospective review of a consecutive series of adult patients with Crowe type IV DDH who underwent THA with porous TM pads and femoral shortening osteotomy at our institution. Written informed consent was obtained from all patients when they came back for follow-up. Patients were identified from a senior surgeon’s database between May 2011 and April 2018. The inclusion criteria encompassed preoperative radiographic evidence of Crowe type IV DDH, treatment with THA using a porous TM pad, femoral shortening osteotomy with a minimum follow-up of 3 years, and records of patient-reported outcomes (Harris Hip Score and Western Ontario and McMaster Universities Arthritis Index (WOMAC)) both preoperatively and postoperatively.

### Surgical technique

**Fig. 1:**
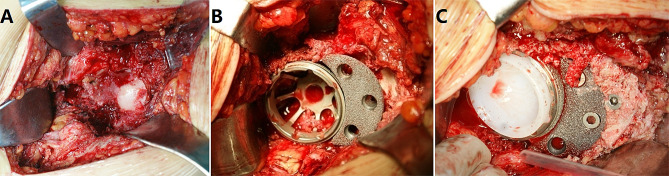
Intraoperative surgical picture of acetabular reconstruction. (**A**) The true acetabulum was meticulously debrided and reamed. (**B**) Autologous bone was applied to the acetabulum combined with uncemented porous tantalum acetabular pads for acetabular reconstruction. (**C**) The tantalum pad was impacted into the acetabulum, and fixation was performed using three cancellous screws

Preoperative surgical planning and template measurements were performed. All surgeries were performed using a posterolateral approach, with the patient in the lateral decubitus position. The femoral head was resected after hip dislocation. Extended trochanteric osteotomy was then performed in the hips, if necessary. The false and true acetabulae were identified after removing the obscuring osteophytes. After confirming the rotational centre, the true acetabulum was meticulously debrided and reamed to expose the robust, vascularized bone. When the acetabular has poor bone mass and provides less than 70% bone coverage of the acetabular component, we choose the acetabular component more than 48 mm. Simultaneously, autologous and artificial bone substitutes were then applied to the acetabulum combined with uncemented porous tantalum acetabular pads (TM Acetabular Revision System; Zimmer) for acetabular reconstruction. The tantalum pad was then impacted into the acetabulum, and fixation was performed using two to four cancellous screws. An appropriately sized acetabular cup was precisely implanted (Fig. 1). Fluoroscopic assessment of position and stability was performed intraoperatively. The hips were subjected to subtrochanteric osteotomy, and the femur was prepared by broaching the proximal region of the femoral component. The transverse shortening osteotomy level was identified as 1 cm distal to the lesser trochanter, and a longitudinal mark was made on the femur as a reference to reestablish femoral rotation after osteotomy. Subsequently, a transverse osteotomy was performed, and the trial S-Rom prosthesis was inserted into the proximal femur to confirm the appropriate size of the selected femoral component. Following osteotomy, hip range of motion and stability were evaluated, and trial implants were adjusted accordingly. The cylindrical segment of the femur removed during shortening osteotomy was split into two or three segments and used as onlay grafts to reinforce the osteotomy site secured with a titanium wire. The patients were instructed to limit their weight-bearing activities and adhere to posterior hip precautions for 12 weeks after surgery.

### Outcome evaluation

All patients underwent postoperative follow-up at 1, 3, and 6 months and annually thereafter. At each visit, functional outcomes were assessed using the WOMAC [19], Harris Score [20], and SF-12 score [21]. Radiographic evaluation was performed at each visit using standard anteroposterior radiographs of the pelvis and full-length radiographs of the lower extremities. Osteolysis of the acetabulum and femur was performed as described previously [22, 23]. The criteria described by Engh et al. were used to assess femoral implant loosening [24].

### Statistical analysis

SPSS 26.0 (IBM Corp., Armonk, NY, USA) was used for all statistical analyses. Demographic data are presented as mean and range values, and percentages are used to describe categorical variables. Data were analyzed using Student’s t-test and are presented as the mean ± standard error of the mean. Kaplan-Meier survival analysis with 95% confidence interval (CI) was used to evaluate overall survival of the acetabular reconstruction and THA with failure defined as revision for any cases. Statistical significance was set at *P* < 0.05.

## Results

A total of 32 hips in 28 patients who underwent surgery between May 2011 (when our use of tantalum pads began) and April 2018, were included in the final analysis. Basic characteristics of the patients, preoperative and postoperative radiographs, and porous TM pad information were collected and reviewed. The average operation time was 176.5 min and the mean blood loss was 224 mL. The mean hospital stay was 7.5 days (Table 1).

**Table 1:** Basic characteristics of the included patients

Parameters	Outcomes
Inclunded cases (n, hips)	*n* = 32
Age (years)	48.4 ± 10.5
Gender (male/female)	8/24
Side (right/left)	17/15
BMI (kg/m^2^)	23.2 ± 4.3
Preoperative Harris hip score	58.4 ± 10.6
Leg length discrepancy, cm	4.6 ± 1.1
Mean pads diameter, mm	55.8 ± 5.4
Hospital stay (day)	7.5 ± 2.8
Operative time (min)	176.5 ± 31.6
Intraoperative blood loss (mL)	224.6 ± 89.6
Acetabular components (Median, mm)	52 (48 to 56)
Follow up (months)	74.3 ± 16.8

**Fig. 2:**
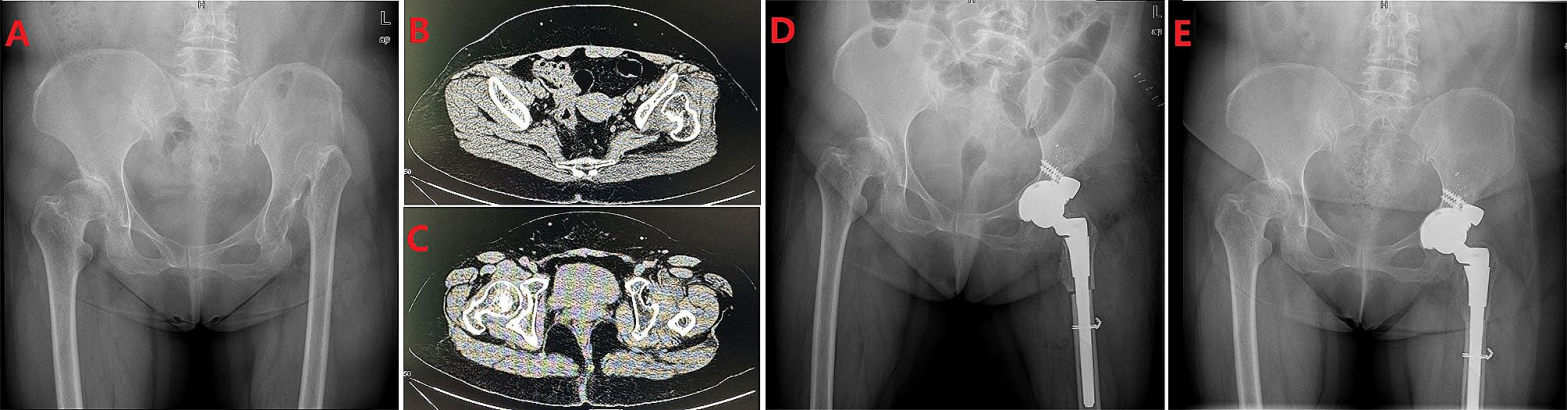
Pre- and postoperative radiographs of a woman with left Crowe type IV DDH. (**A**) Preoperative pelvic radiograph of a 60-year-old female patient with left Crowe type IV developmental dysplasia of the hip (DDH). (**B**) CT image shows the position of the left false acetabulum. (**C**) CT image shows bilateral true acetabulum position. (**D**) Postoperative pelvis radiograph demonstrating left acetabular reconstruction using a TM pads, and femoral shortening osteotomy on each side. (**E**) six years post-surgery, the pelvic X-ray revealed no evidence of loosening or absorption on the acetabular side and complete osseous integration on the femoral side without any subsidence

**Fig. 3:**
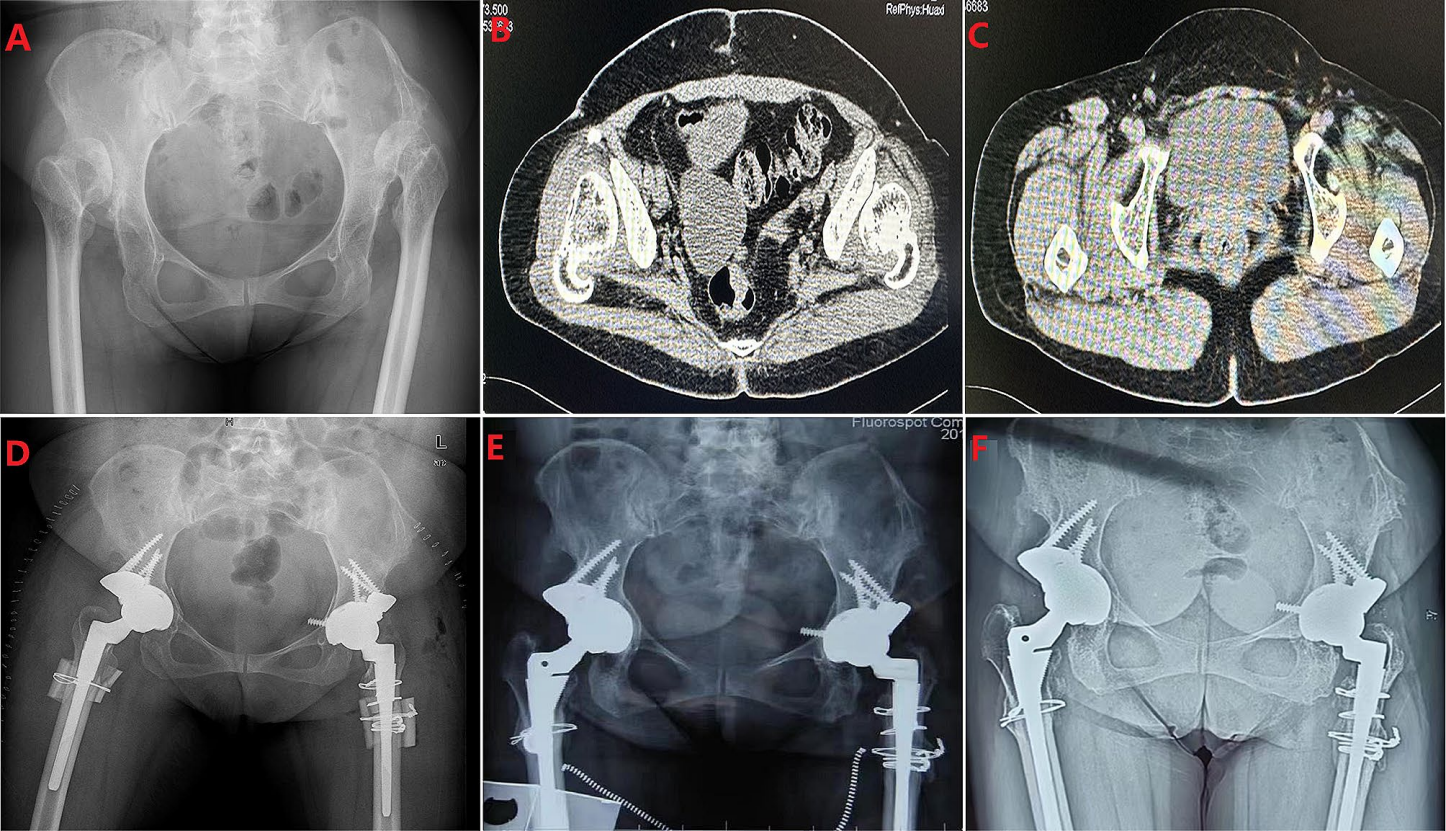
Pre- and postoperative radiographs of a woman with Crowe type IV DDH. (**A**) Preoperative pelvis radiograph of a 44-year-old woman with bilateral Crowe type IV DDH. (**B**) CT image shows the position of the bilateral false acetabulum. (**C**) CT image shows the bilateral true acetabulum position. (**B**) Postoperative pelvis radiograph demonstrating bilateral acetabular reconstruction using a tanium mesh cup, cemented liner, and femoral shortening osteotomy on each side. (**C**) Three years post-surgery, the pelvic X-ray revealed no evidence of either hip socket loosening or absorption.(**D**)After a post-surgical period of 11 years, the pelvic X-ray revealed no evidence of loosening or absorption on the acetabular side and complete osseous integration on the femoral side without any subsidence

At a mean follow-up of 74.3 months (42 to 132 months), the pelvic radiographs displayed that the mean hip centre position was 2.92 ± 0.51 cm horizontally and 2.12 ± 0.48 cm vertically, and the mean acetabular inclination was 37.8 ± 7.2° (Table 2). Two patients who underwent single hip reconstruction (6.25%) had severe limping, two (6.25%) had moderate limping, and 13 (40.6%) had mild postoperative limping (Figs. 2 and 3). The Harris Hip Score improved from 58.4 ± 10.6 preoperatively to 85.6 ± 8.9 at the last follow-up (*P* < 0.01). The Mean WOMAC scores were 86.5 ± 10.2 for pain, 87.3 ± 12 for stiffness, and 85.4 ± 11.6 for function with a score of 90.4 ± 7.6 for patient satisfaction. The mean scores of patient satisfaction were 90.4 ± 7.6, indicating a good level of satisfaction regarding pain relief, function, and recreational activities (Table 2). SF-12 physical summary score was 41.8 ± 5.6 and SF-12 mental summary score was 51.6 ± 5.4 (Table 2).

**Table 2:** Clinical and radiological results

Variable	Outcomes
Hip centre horizontally (cm)	2.92 ± 0.51
Hip centre vertically (cm)	2.12 ± 0.48
Acetabular inclination (°)	37.8 ± 7.2
Leg length discrepancy (cm)	1.14 ± 1.04
Postoperative Limping Levels Severe, n (%) Moderate, n (%) Mild, n (%) None, n (%)	2(6.25%) 2(6.25%) 13(40.63%) 15(46.88%)
Harris hip score	85.6 ± 8.9
WOMAC* scores Pain Stiffness Function	86.5 ± 10.2 87.3 ± 12.4 85.4 ± 11.6
SF-12* physical summary score SF-12* mental summary score	41.8 ± 5.6 51.6 ± 5.4
Patient satisfaction score	90.4 ± 7.6

*WOMAC and SF-12 scores are normalized to a range of 0 to 100 points, with 0 being worst and 100 being best. Satisfaction score: 0 is worst, 100 is best

No intraoperative complications occurred during the acetabular placement in any patient. However, intraoperative femoral fracture occurred in two patients who underwent transverse subtrochanteric osteotomy. Six cases of postoperative intramuscular thrombosis were observed, and two patients experienced sciatic nerve palsy after surgery. However, both cases resolved with no residual deficits at the 6-month follow-up visit. Additionally, one case of subtrochanteric osteotomy bone nonunion and another of postoperative prosthesis dislocation, both of which underwent secondary surgery. Two patients developed painful aseptic loosening of the acetabular component and TM pads. The previous large-diameter cup was replaced with pads of appropriate dimensions and contours, resulting in two patients undergoing allogeneic granular bone grafts in conjunction with the replacement procedure. Postoperative follow-up revealed favourable outcomes. No pulmonary emboli, deep venous thromboses, or cardiac or cerebrovascular complications were observed during the follow-up period (Table 3).

**Table 3:** Complications occorrence

Complications	hips, *n*
Pulmonary embolism	None
Deep vein thrombosis	None
Periprosthesis infection	None
Cardio-cerebrovascular complications	None
Component loosening	None
Intraoperative fracture	2
Dislocation	1
Nonunion	1
Leg length discrepancy	1
Nerve palsy	2
Intermuscular vein thrombosis	6
stem aseptic loosening	1
Cup and pads migration	1
Hip revision	2

**Fig. 4:**
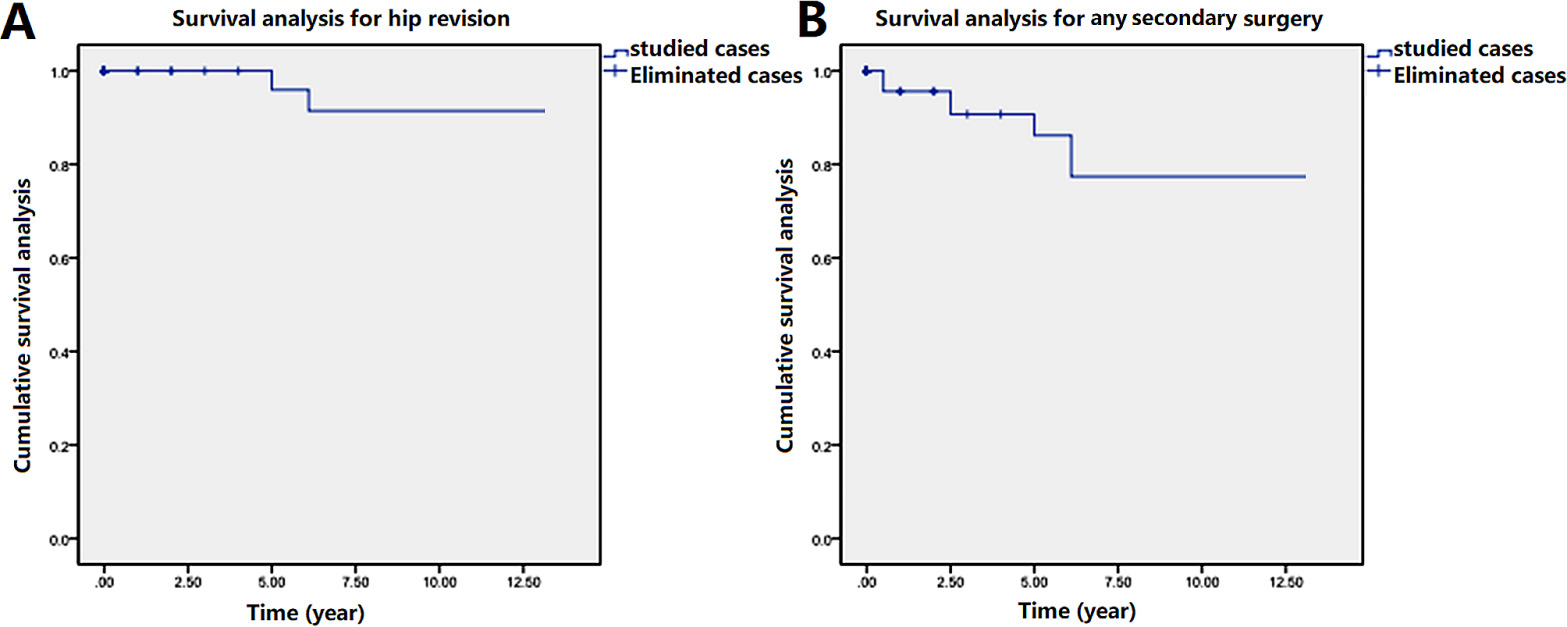
Survival analysis based on the cases of hip revision (**A**) and any secondary surgery (**B**)

TM construct Kaplan-Meier survivorship at a mean follow-up of 6.2 years, the TM construct survivorship due to all-cause failure was 90.6% at 5 years with 3 hips at risk, 87.5% at 10 years with 4 hips at risk. The survivorship due to failure from aseptic loosening was 96.9% at 5 years with 1hips at risk and 93.75% at 10 years with 2 hips at risk. (Figs. 4)

## Discussion

The technique of using porous tantalum TM pads for acetabular reconstruction during complex revision THA has been providing increasing evidence of excellent early to long-term outcomes as reported in the published series [25–29]. We have been utilising this technology for several years to manage Crowe type IV DDH with acetabular insufficiency.

THA for Crowe type IV DDH is widely acknowledged to have a higher incidence of complications and failure rates than routine THA for primary osteoarthritis [10]. The preoperative assessment of the acetabular and proximal femoral anatomy, in conjunction with surgical techniques and expertise, are pivotal factors for achieving successful outcomes (Table 4). The current retrospective study presents the mid-term outcomes of a series of patients. with Crowe type IV DDH who underwent THA using a tantalum pad and subtrochanteric femoral shortening osteotomy.

**Table 4:** Review the literature on treatment techniques of crowe type IV DDH

Author	Cases (*n*, hips)	Follow-up	Acetabulum Techniques	Femur technique	Success rate (%)	Harris scores
Ors C [2]	127	8.4 years	Trilogy IT acetabular Component	Wagner cone stem + TSO	94.5%	94.2 ± 6.9
Caylak R [3]	67	> 10 years	Cementless acetabular component	Ceramic- on-ceramic bearing + TSO	94%	94.1 ± 8.1
Ma HY [6]	116	71.3 months	Pinnacle acetabular cup	Ceramic-on-ceramic bearing + modular S-ROM stem + TSO	98.3%	91.3 ± 4.2
Krych AJ [9]	27	4.8 years	Porous-coated acetabular component	Extensively porous-coated stems/S-ROM + TSO	77.8%	89
Farrell CM [30]	28	8–15 years	Uncemented/cemented acetabular component + FHSA	Not reported	82.1%	not reported
Zhao HY [31]	50	2 years	Porous-coat acetabular components	TSO	98%	96.5 ± 9.6
Pei L [32]	12	42.2 months	Allofit biologic acetabulum components + FHSA	ML wedge femoral stem	91.7%	89.7 ± 3.9

TSO: transverse subtrochanteric osteotomy; FHSA: ipsilateral femoral head structural autograf

Reconstruction of acetabulum in Crowe type IV DDH is always the challenges for surgeons, for example, the acetabulum was undeveloped or poorly developed, acetabular bone mass is limited, acetabular component placed in the true acetabulum position has poor coverage of the acetabular roof and severe bone defect. Reconstructing the acetabulum to its true anatomical position has previously been demonstrated to enhance hip biomechanics in cases of DDH [33, 34]. Many different strategies have been used to reconstruct the acetabulum. These include autogenous femoral head transplantation, impacted bone grafting, and the use of metal cups with particle bone grafting [17]. Our previous study used porous-coat acetabular components and transverse subtrochanteric osteotomy to manage patients with Crowe type IV DDH. The clinical outcomes of our initial study were encouraging, although acetabular reconstruction was difficult in several patients [31]. In the current study, stable acetabular component fixation was obtained using tantalum metal blocks to reconstruct dysplastic acetabular defects exceeding 30%. In our mid-term study, no obvious signs of implant migration or subsidence were observed.

Porous tantalum, also known as trabecular metal, is a popular choice for managing bone loss in acetabular reconstruction due to its exceptional mechanical properties. Firstly, tantalum exhibits a higher porosity, approximately two to three times that of cobalt, chromium, and titanium, thereby promoting significant bone and fibrous ingrowth potential [35, 36]. Secondly, the elastic modulus of porous tantalum (also known as trabecular metal) closely resembles that of trabecular bone. Consequently, porous tantalum facilitates the physiological transfer of load from the implant to the host bone, minimizes stress-shielding effects, and preserves the bone stock [37]. Thirdly, biomechanical testing demonstrated that porous tantalum possesses a high coefficient of friction, which imparts superior implant stability compared to that of traditional cementless implants [38]. The attainment of intraoperative stability of the acetabular components is crucial for achieving successful mid-term outcomes [39]. Hence, porous tantalum metal has been used for acetabular bone defect reconstruction in hip revision for some time. However, the application of porous tantalum TM pads for acetabular reconstruction in patients with Crowe type IV DDH has not yet been widely reported.

The available reconstructive techniques for DDH are extensive, and this series cannot compare our results with those of alternative approaches. Other techniques for reconstructing DDH involve the utilisation of acetabular reinforcement devices in conjunction with structural bone grafting, autogenous femoral head grafting, and alternative metal cups combined with allogenic structural bone grafting. However, graft healing and stability are crucial technical factors. The assimilation of an autogenous femoral head bone graft into the pelvis occurs slowly and incompletely, rendering it susceptible to collapse and resorption [30]. The other reconstruction methods, such as incarcerated and granular bone grafting, fail to provide sufficient initial stability for the acetabulum alone for rapid absorption, thereby compromising long-term stability [32]. The stability of other bone grafts fixed with metal mesh and cups attached with bone cement can be deemed sufficient; however, their long-term survival rate is unsatisfactory, and the incidence of aseptic loosening is high [40]. In the current study, we used porous TM pads for acetabular reconstruction and achieved promising mid-term follow-up clinical results with a 6.2-year success rate of 93.75% and a mean Harris score of 85.6. The results of our study were comparable to the results obtained from previous studies (Table 4).

Our study had several limitations. The sample size was relatively small, which limited the ability of our dataset to draw definitive conclusions regarding patient outcomes. The technique described in this study was initially used at our institution a decade ago. The initial number of treated patients was lower than the current practice volume. The small number of patients treated in the past limited the number of patients who met the length of follow-up required for inclusion in this study. Future studies should incorporate a large sample size of patients with adequate follow-up duration. The patients included in our study were relatively young, with a mean age of 48.4 years (range 34–85) at the time of the revision surgery. Consequently, acknowledging that our findings are limited to this specific age group and may not be readily extrapolated to older populations is important. Additionally, we did not investigate the relationship between the spine and pelvis, which is another limitation. Despite the follow-up period spanning from 4 to 10 years (with an average duration of 6.2 years), a significant portion of the data was presented during the mid-term assessment, highlighting the need for future investigations to explore long-term outcomes.

## Conclusion

This study demonstrates that the implementation of porous TM pads for acetabular reconstruction in THA for Crowe type IV DDH yields favourable outcomes at mid-term follow-up, thereby potentially serving as a viable therapeutic option for patients affected by this severe deformity.

## Data Availability

The datasets used and/or analyzed during the current study are available from the corresponding author on reasonable request.

## References

[1] Gharanizadeh K, Mahmoudi M, Shiva F, Ghazavi M, Abolghasemian M (2023). Assessing Leg length discrepancy is necessary before arthroplasty in patients with Unilateral Crowe type IV hip dislocation. Clin Orthop Relat Res.

[2] Ors C, Caylak R, Togrul E (2022). Total hip arthroplasty with the Wagner cone femoral stem in patients with Crowe IV Developmental Dysplasia of the hip: a retrospective study. J Arthroplasty.

[3] Caylak R, Ors C, Togrul E (2021). Minimum 10-Year results of Cementless Ceramic-On-Ceramic total hip arthroplasty performed with transverse Subtrochanteric Osteotomy in Crowe Type IV hips. J Arthroplasty.

[4] Harris WH (1969). Traumatic arthritis of the hip after dislocation and acetabular fractures: treatment by mold arthroplasty. An end-result study using a new method of result evaluation. J Bone Joint Surg Am.

[5] Du YQ, Zhang B, Sun JY, Ma HY, Shen JM, Ni M, Zhou YG (2021). The variation of the Pelvis in Unilateral Crowe Type IV Developmental Dysplasia of the hip. Orthop Surg.

[6] Ma HY, Lu Q, Sun JY, Du YQ, Shen JM, Gao ZS, Lu SB, Zhou YG (2020). One-stage total hip arthroplasty with modular S-ROM stem for patients with bilateral Crowe type IV Developmental Dysplasia. Orthop Surg.

[7] Tao K, Wang SC, Ma XY, Shao L, Di ZL, Huang ZY (2023). Three-dimensional femur morphology analysis for the optimal location of subtrochanteric osteotomy with an implanted Wagner cone stem in total hip arthroplasty for Crowe type IV developmental dysplasia of the hip. J Orthop Surg Res.

[8] Miyazaki T, Shimizu T, Ohura H, Katayama N, Iwasaki N, Takahashi D (2023). Total hip arthroplasty with femoral shortening osteotomy using polished cemented stem vs. modular cementless stem in patients with Crowe type IV developmental dysplasia of the hip. Arch Orthop Trauma Surg.

[9] Krych AJ, Howard JL, Trousdale RT, Cabanela ME, Berry DJ (2009). Total hip arthroplasty with shortening subtrochanteric osteotomy in Crowe type-IV developmental dysplasia. J Bone Joint Surg Am.

[10] Akıncı O, Turgut A (2021). Long-term results of total hip arthroplasty with Step-Cut Osteotomy in Crowe Type IV dysplastic hips. Indian J Orthop.

[11] Kilicoglu OI, Turker M, Akgul T, Yazicioglu O (2013). Cementless total hip arthroplasty with modified oblique femoral shortening osteotomy in Crowe type IV congenital hip dislocation. J Arthroplast.

[12] Karachalios T, Hartofilakidis G (2010). Congenital hip disease in adults: terminology, classification, pre-operative planning and management. J bone Joint Surg Br Volume.

[13] Erdemli B, Yilmaz C, Atalar H, Guzel B, Cetin I (2005). Total hip arthroplasty in developmental high dislocation of the hip. J Arthroplasty.

[14] Yang S, Cui Q (2012). Total hip arthroplasty in developmental dysplasia of the hip: review of anatomy, techniques and outcomes. World J Orthop.

[15] Delimar D, Bicanic G, Korzinek K (2008). Femoral shortening during hip arthroplasty through a modified lateral approach. Clin Orthop Relat Res.

[16] Bicanic G, Barbaric K, Bohacek I, Aljinovic A, Delimar D (2014). Current concept in dysplastic hip arthroplasty: techniques for acetabular and femoral reconstruction. World J Orthop.

[17] Wang Y, Wang M, Li C, Nakamura Y, Deng L, Yamako G, Chosa E, Pan C (2022). Biomechanical effect of metal augment and bone graft on cup stability for acetabular reconstruction of total hip arthroplasty in hip dysplasia: a finite element analysis. BMC Musculoskelet Disord.

[18] Alqwbani M, Wang Z, Wang Q, Li Q, Yang Z, Kang P (2022). Porous tantalum shell and augment for acetabular defect reconstruction in revision total hip arthroplasty: a mid-term follow-up study. Int Orthop.

[19] Bellamy N, Buchanan WW, Goldsmith CH, Campbell J, Stitt LW (1988). Validation study of WOMAC: a health status instrument for measuring clinically important patient relevant outcomes to antirheumatic drug therapy in patients with osteoarthritis of the hip or knee. J Rheumatol.

[20] Mahomed NN, Arndt DC, McGrory BJ, Harris WH (2001). The Harris hip score: comparison of patient self-report with surgeon assessment. J Arthroplast.

[21] Ware J, Kosinski M, Keller SD (1996). A 12-Item short-form Health Survey: construction of scales and preliminary tests of reliability and validity. Med Care.

[22] DeLee JG, Charnley J. Radiological demarcation of cemented sockets in total hip replacement. Clin Orthop Relat Res. 1976(121):20–32. 991504

[23] Gruen TA, McNeice GM, Amstutz HC. Modes of failure of cemented stem-type femoral components: a radiographic analysis of loosening. Clin Orthop Relat Res. 1979(141):17–27. 477100

[24] Engh CA, Bobyn JD, Glassman AH (1987). Porouscoated hip replacement. The factors governing bone ingrowth, stress shielding, and clinical results. J bone Joint Surg Br Volume.

[25] Eachempati KK, Malhotra R, Pichai S (2018). Results of trabecular metal augments in Paprosky IIIA and IIIB defects. Bone Joint J.

[26] Abolghasemian M, Tangsataporn S, Sternheim A (2013). Combined trabecular metal acetabular pads and augment for acetabular revision with substantial bone loss: a mid-term review. Bone Joint J.

[27] Jenkins DR, Odland AN, Sierra RJ, Hanssen AD, Lewallen DG. Minimum fve year outcomes with porous tantalum acetabular cup and augment construct in complex revision total hip arthroplasty. J Bone Joint Surg Am 2017; 99-A:e49. 10.2106/JBJS.16.00125 28509833

[28] Löchel J, Janz V, Hipf C, Perka C, Wassilew GI (2019). Reconstruction of acetabular defects with porous tantalum padss and augments in revision total hip arthroplasty at ten-year follow-up. Bone Joint J.

[29] Konan S, Duncan CP, Masri BA, Garbuz DS (2016). Porous tantalum uncemented acetabular components in revision total hip arthroplasty: a minimum ten-year clinical, radiological and quality of life outcome study. Bone Joint J.

[30] Farrell CM, Berry DJ, Cabanela ME (2005). Autogenous femoral head bone grafts for acetabular deficiency in total-hip arthroplasty for developmental dysplasia of the hip: long-term effect on pelvic bone stock. J Arthroplasty.

[31] Zhao HY, Kang PD, Shi XJ, Zhou ZK, Yang J, Shen B, Pei FX (2019). Effects of total hip arthroplasty on Axial Alignment of the Lower Limb in patients with Unilateral Developmental Hip Dysplasia (Crowe type IV). J Arthroplasty.

[32] Pei L, Zhou X, Wu Y, Liu Y, Xue Y, Meng F, Liu B (2022). [Short-term effectiveness of structural bone graft and total hip arthroplasty through direct anterior approach in lateral decubitus position for Crowe type III and IV developmental dysplasia of the hip]. Zhongguo Xiu Fu Chong Jian Wai Ke Za Zhi.

[33] Oinuma K, Tamaki T, Miura Y, Kaneyama R, Shiratsuchi H (2014). Total hip arthroplasty with subtrochanteric shortening osteotomy for Crowe grade 4 dysplasia using the direct anterior approach. J Arthroplast.

[34] Hasegawa Y, Iwase T, Kanoh T, Seki T, Matsuoka A (2012). Total hip arthroplasty for Crowe type developmental dysplasia. J Arthroplast.

[35] Bobyn JD, Stackpool GJ, Hacking SA (1999). Characteristics of bone ingrowth and interface mechanics of a new porous tantalum biomaterial. J Bone Joint Surg Br.

[36] Levine BR, Sporer S, Poggie RA (2006). Experimental and clinical performance of porous tantalum in orthopedic surgery. Biomaterials.

[37] Meneghini RM, Ford KS, McCollough CH (2010). Bone remodeling around porous metal cementless acetabular components. J Arthroplasty.

[38] Meneghini RM, Meyer C, Buckley CA (2010). Mechanical stability of novel highly porous metal acetabular components in revision total hip arthroplasty. J Arthroplasty.

[39] Sporer SM, Paprosky WG (2006). The use of a trabecular metal acetabular component and trabecular metal augment for severe acetabular defects. J Arthroplasty.

[40] Maruyama M, Wakabayashi S, Ota H, Tensho K (2017). Reconstruction of the shallow Acetabulum with a combination of Autologous Bulk and Impaction Bone Grafting fixed by cement. Clin Orthop Relat Res.

